# Machine Learning Approaches for Monitoring of Tool Wear during Grey Cast-Iron Turning

**DOI:** 10.3390/ma15124359

**Published:** 2022-06-20

**Authors:** Maciej Tabaszewski, Paweł Twardowski, Martyna Wiciak-Pikuła, Natalia Znojkiewicz, Agata Felusiak-Czyryca, Jakub Czyżycki

**Affiliations:** 1 Institute of Applied Mechanics, Faculty of Mechanical Engineering, Poznan University of Technology, 3 Piotrowo St., 60-965 Poznań, Poland; maciej.tabaszewski@put.poznan.pl; 2 Institute of Mechanical Technology, Faculty of Mechanical Engineering, Poznan University of Technology, 3 Piotrowo St., 60-965 Poznań, Poland; pawel.twardowski@put.poznan.pl (P.T.); natalia.w.znojkiewicz@doctorate.put.poznan.pl (N.Z.); agata.z.felusiak@doctorate.put.poznan.pl (A.F.-C.); jakub.r.czyzycki@doctorate.put.poznan.pl (J.C.)

**Keywords:** machine learning, tool wear identification, diagnostic system

## Abstract

The dynamic development of new technologies enables the optimal computer technique choice to improve the required quality in today’s manufacturing industries. One of the methods of improving the determining process is machine learning. This paper compares different intelligent system methods to identify the tool wear during the turning of gray cast-iron EN-GJL-250 using carbide cutting inserts. During these studies, the experimental investigation was conducted with three various cutting speeds *vc* (216, 314, and 433 m/min) and the exact value of depth of cut *a_p_* and federate *f*. Furthermore, based on the vibration acceleration signals, appropriate measures were developed that were correlated with the tool condition. In this work, machine learning methods were used to predict tool condition; therefore, two tool classes were proposed, namely usable and unsuitable, and tool corner wear *VBc* = 0.3 mm was assumed as a wear criterium. The diagnostic measures based on acceleration vibration signals were selected as input to the models. Additionally, the assessment of significant features in the division into usable and unsuitable class was caried out. Finally, this study evaluated chosen methods (classification and regression tree, induced fuzzy rules, and artificial neural network) and selected the most effective model.

## 1. Introduction

The interest in computer technics is evident in advanced approaches to engineering issues. Furthermore, to apply intelligent systems is to improve the determined process, achieving the required quality. According to the Fourth Industrial Revolution concepts, the intelligent systems correlated with computer algorithms, leading to improved efficiency of technological processes, reducing costs, or keeping downtime to an absolute minimum. The sophisticated methods and informatics tools are used as assistance systems in shaping materials by machining [1,2,3]. More and more satisfactory results are noticeable due to artificial intelligence (AI) application, where the algorithms are related with “learning” nonlinear dependencies between input and output data. For example, one of the primary AI applications in machining is tool wear or surface roughness prediction based on phenomena occurring in machining, such as cutting forces, vibrations, or acoustic emission [4,5,6,7,8]. The correlation between those quantities with tool wear allows tool identification in real time and simultaneously eliminates optional downtimes. One of the subsets of artificial intelligence is machine learning. Its straightforward structure allows for the design of clear rules to obtain an independent expert system. Classical regression models are ineffective in some engineering issues, and the correlation coefficient is too low to implement diagnostic inference. A more complex model, such as machine learning, is therefore valid in solving complicated issues. The dynamic development of new technologies, including material engineering, enables the optimal material choice that fulfills high requirements. For example, the aviation industry nowadays relies on materials with improved properties, such as nanocomposites and superalloys. As a result, more and more elements are made of non-ferrous materials, alloys based on aluminum, nickel, or copper. The fact remains that most foundries specialize in producing casts made of iron alloys. However, cast iron’s demands on their mechanical properties still find usage in areas such as the automotive or railway industries. Of the many types of cast iron, gray iron is used the most [9]. This material offers a good cast-ability, competitive strength-to-cost ratio, and high machinability. However, the possibility of gray cast-iron applications in challenging areas demands the characteristic morphology of the graphite phase. The matrix of gray cast iron consists of a soft ferrite phase and lamellar pearlite phase [10,11]. However, mechanical properties depend on the chemical composition, the type of matrix and form of graphite (flake, compacted, nodular), or heat and cooling cycle [12,13]. Furthermore, due to the specific distribution of graphite in gray iron microstructure, the tool wear mechanism during machining has been a research topic in recent years [14,15,16,17]. In work [18], Guizhao Tu et al. investigated tool wear behavior during high-speed dry turning of gray cast iron with Sialon tools. They compared two Sialon inserts: one of them with higher Vickers hardness (sample A) and the other with higher fracture toughness (sample B). They noticed that value of flank wear width (*VB*) of sample A increased faster in lower cutting speed (300 m/min) than sample B, but in higher cutting conditions, the sample A tool life was longer. The authors also recognized that the dominant wear mechanism changed with increasing cutting speed from abrasion to adhesion. Paolo Fiorini et al. explored the influence of the protective built-up layer (BUL) formation in PCBN tools on GG25 gray cast-iron turning [19]. In this work, the authors observed BUL mainly consisting of Mn and S, formed in lower (250 m/min) and higher cutting speed (750 m/min). They also noticed that this protective layer forms over an area on the rake and flank face in lower cutting speed, but its location on the tool is coupled with cutting temperature and chip length. Moreover, at 750 m/min speed, they observed a higher protecting layer in maximum temperature area and reduced wear rates, and the crater wear was only detected in lower cutting speed. The built-up layer could be the key to tool protection and tool life increase in gray cast-iron machining. Due to interests growth in intelligent systems in engineering issues, most works nowadays focus on applied computer techniques to improve machining, which is directly connected with tool condition monitoring systems (TCMs) [20,21,22]. In order to identify tool wear, various kinds of machine learning models, such as decision trees (DT), artificial neural network (ANN), support vector machine (SVM), or hidden Markov model (HMM), are applied to create an effective diagnostic system [23,24,25]. In [26] work, Aissa Laouissi et al. investigated the optimization procedure during gray cast-iron turning with artificial neural network approach (ANN), the response surface methodology (RSM), and genetic algorithm (GA). They developed a surface roughness, cutting force, and cutting power prediction model based on cutting parameters (used as inputs to the model). The ANN models provide better results (5.89% error) than the RSM model (14.73% error) for surface roughness prediction. The application of genetic algorithm optimization also enabled finding the best cutting parameters to lead to better surface quality and a minimum cutting force (*v_c_* = 299.525–512.571 m/min, *f* = 0.8–0.121 mm/rev, *a_p_* = 0.251–0.586 mm). Other studies are focused on prediction using cutting parameters and vibration signals. such as in ref. [27]. In this research, Johnny Herwan et al. proposed a surface roughness prediction model based on ANN during dry gray cast-iron turning. They obtained surface roughness prediction with an average error below 8%. However, computer techniques are also used to predict the mechanical properties of engineering materials. For example, Masato Shirai et al. [28] used the deep neural network (DNN) to predict mechanical properties of gray cast iron based on chemical compositions, including trace elements. This work developed the adequate tensile strength and Brinell hardness prediction model with 5.12% mean absolute error for tensile strength and 4.18% for hardness. The obtained results of several authors confirmed the validity of intelligent systems in engineering issues. This kind of computer technique supports optimization of the machining process.

The following study investigates the tool identification model during the turning of gray cast-iron EN-GJL-250 using carbide cutting inserts. During these studies, the experimental investigation was conducted with three various cutting speeds *v_c_* (216, 314, and 433 m/min) and the exact value of depth of cut *a_p_* and federate *f*. Further, a tool wear prediction model was developed based on machine learning and vibration acceleration signals.

## 2. Materials and Methods

**Figure 1:**
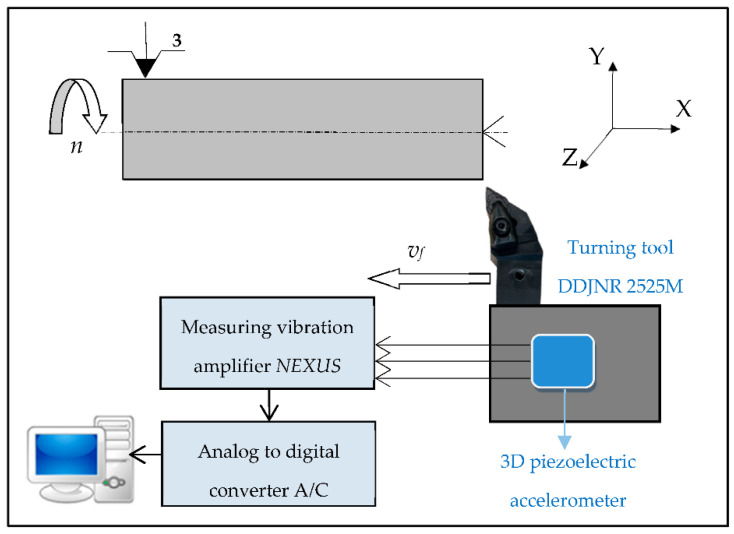
Scheme of experimental set up.

The experimental study of grey cast-iron EN-GJL-250 turning was carried out on TUR 560E manual lathe with three different cutting speeds *v_c_*. During the investigation, the cutting tool with cemented carbide inserts (DNMG 15 06 08-WF 3210, Sandvik Coromant, Sandviken, Sweden) was used in the machining process. The triaxial piezoelectric charge accelerometer (type 4321 Brüel and Kjær, Nærum, Denmark) was selected to measure vibrations in three independent directions (X,Y,Z) and attached to a tool holder with a screw joint. This accelerometer is suited to measure up 10,000 Hz, with sensitivity 1 pC/ms^−2^. During each turning pass in which the vibration acceleration were measured, these signals obtained during the research were applied to build a diagnostic system. Simultaneously, with the measurement of the vibrations, the tool corner wear *VB_C_* was inspected using a workshop microscope (scale interval 0.01 mm). Based on “Analyzer” software (developed by Maciej Tabaszewki in Poznan, Poland), the vibration charts in time and frequency domain were used to select the diagnostic measures. Figure 1 shows the scheme of the experimental apparatus setup.

The cutting speed *v_c_* was one variable parameter in the tests. The research plan of an experiment is shown in Table 1.

**Figure 2:**
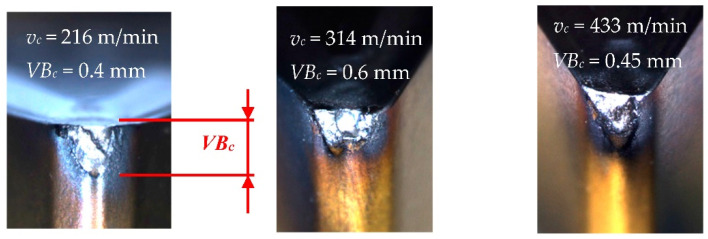
Tool corner wear *VB_C_* after turning with various cutting speed.

The tool corner wear indicator *VB_C_* was measured every few minutes during each test. Each tool wear measurement corresponded to the vibration acceleration signals in three directions (main *Ac*, feed *Af*, and axial direction *Ap*). The signals from the accelerometer were transferred to the measuring vibration amplifier NEXUS and then to the analog-to-digital converter A/C and the desktop. The “Analyzer” software was applied to determine the diagnosis measures of vibration accelerations. The data from the turning process were the foundation for developing a diagnostic system. Figure 2 depicts *VB_C_* the tool corner wearing in various cutting conditions.

**Figure 3:**
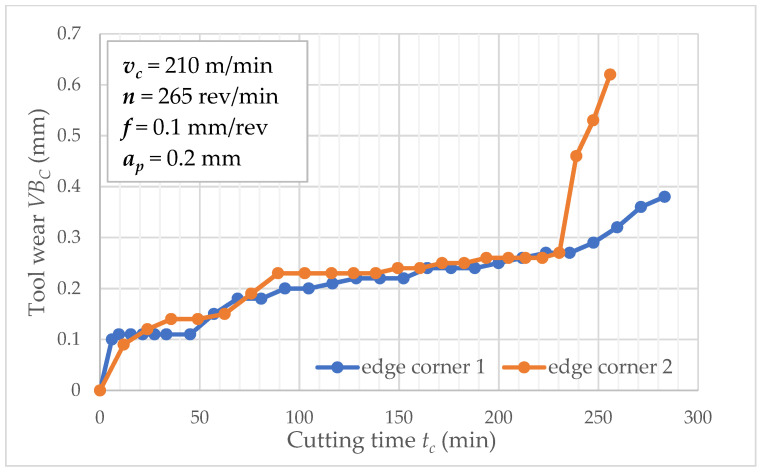
Tool wear *VB_C_* as a function of cutting time *t_c_* (*v_c_* = 210 m/min).

**Figure 4:**
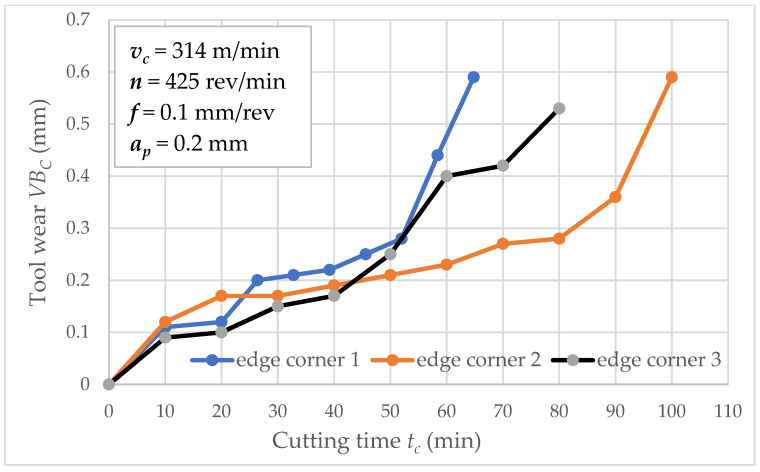
Tool wear *VB_C_* as a function of cutting time *t_c_* (*v_c_* = 314 m/min).

**Figure 5:**
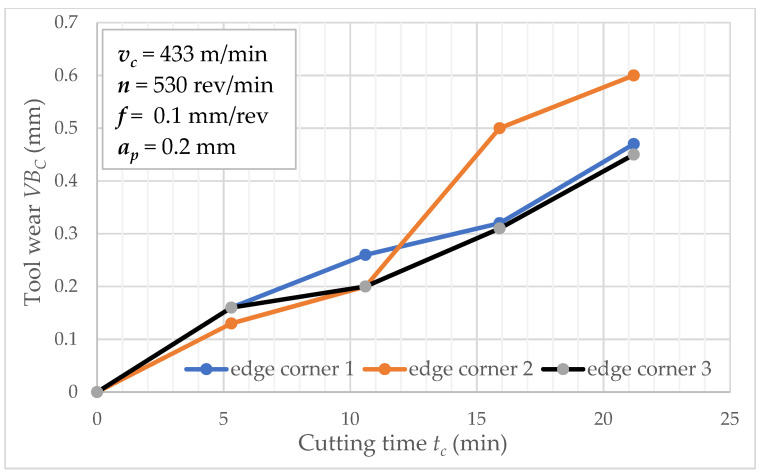
Tool wear *VB_C_* as a function of cutting time *t_c_* (*v_c_* = 433 m/min).

Figure 3 shows tool wear *VB_C_* values in function of cutting time *t_c_* separately for wedge corners 1 and 2. The flow of these functions are similar until *t_c_* = 230 min; then, the difference between corner 1 and 2 is significant. This is a typical phenomenon in the tool wear process because a random factor determines the changes. The other graphs show tool wear as a function of time for cutting speeds 314 and 433 m/min (Figure 4 and Figure 5). In such conditions, three repetitions were carried out for these cutting speeds. As the figures depict, the increase in cutting speed contributes to increased tool wear process intensity, directly affecting the tool life of inserts. Analyzed data related to a series of two tool life tests for feed *f* = 0.1 mm/rev. It is largely known that cutting speed affects the tool life and feed *f* value (at a slower rate). Experimental study of tool life tests was carried out for feed 0.05 and 0.2 mm/rev as analogous to feed *f* = 0.1 mm/rev.

The tool wear criterion was determined in order to indicate the tool life. In addition, the selection of tool wear criterion is not apparent, including even a possibility to indicate several criteria in industrial conditions. The establishment of criteria depends on many factors, primarily on machining form (roughing, finishing), the machining strategy, or the machining process. The geometric criteria are the most accurate and reliable in laboratory conditions. In contrast, the technological and physical criteria that prevail in the industry conditions are set on an ongoing basis according to users’ needs.

As a result of these studies, it was decided to determine the geometric criterion for the *VB_C_* indicator:

the tool wear criterion − *VB_c_* < 0.3 mm

When the tool corner wear of the insert exceeds the 0.3 mm value, it is recognized as unfit and requires replacement. Otherwise, the wedge is classified as capable of further work. 

**Figure 6:**
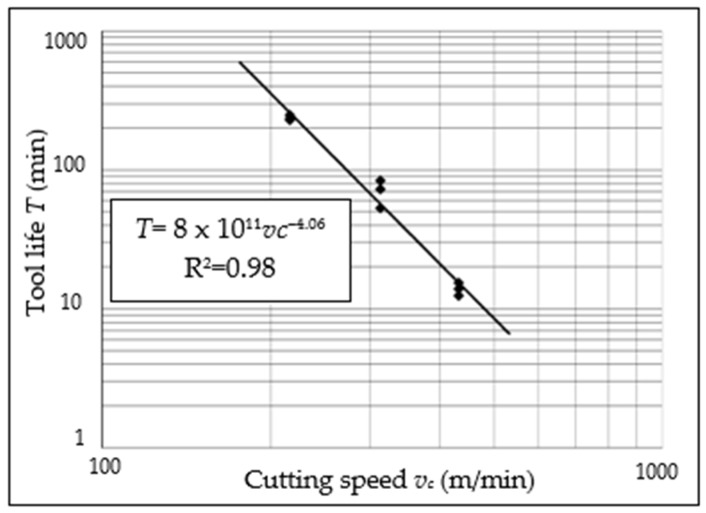
Graphic interpretation of Taylor’s tool life formula (relation between tool life *T* and cutting speed *v_c_*).

The tool life for individual inserts was determined based on an established geometric criterion, and summary results are shown in a double-logarithmic system, specifying Taylor’s tool life formula (Figure 6). The relation between tool life *T* and cutting speed *v_c_* is essential from a practical point of view. It is also possible to select a relevant cutting speed for a productive lifecycle, assuming a tool life. 

It is recognized that cutting speed increases generate a decrease in the value of tool life. However, on the other hand, the efficiency of the machining process is improved. Therefore, it is necessary to obtain the cutting speed in which the tool life and cutting performance will be acceptable.

Using the Formula (1), the cutting time of the tool (tool life *T*) until the tool wear criterion exceeds for particular cutting speed *v_c_* [29]:

(1) $$T=\frac{1\times 10^{12}}{v_{c}^{4.1}}\;\left(\min\right).\;$$

The determined Taylor’s tool life formulas (*T* = *f*(*v_c_*,*f*)) are useful for cutting time prediction and cutting parameters’ correction.

**Figure 7:**
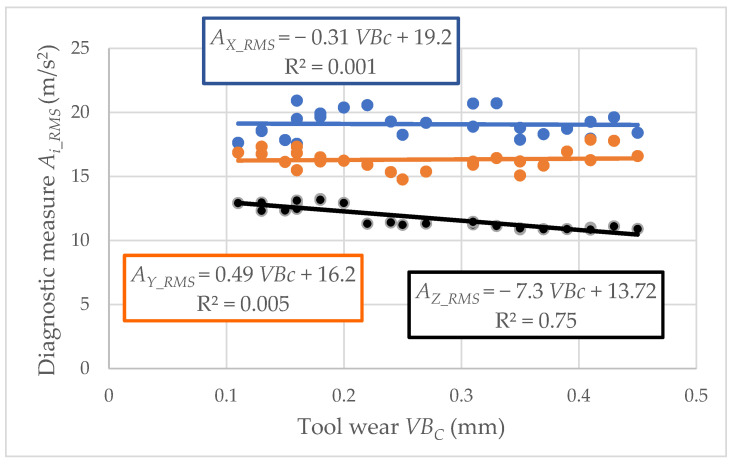
Relation between the vibration acceleration amplitude and tool wear *VB_C_* (cutting speed *v_c_* = 433 m/min; edge 1).

**Figure 8:**
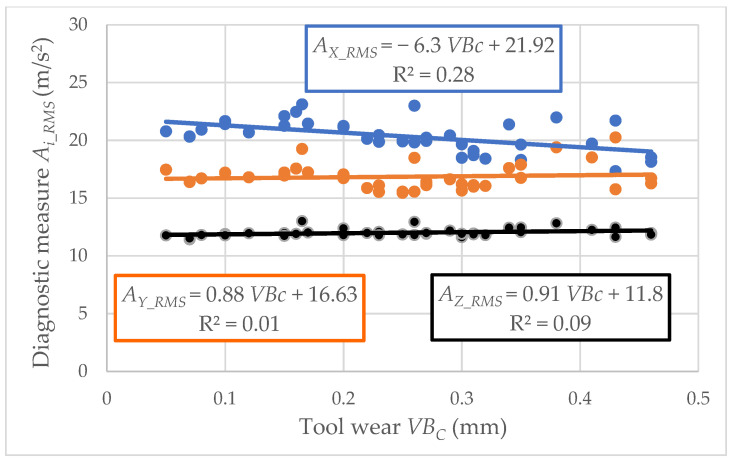
Relation between the vibration acceleration amplitude and tool wear *VB_C_* (cutting speed *v_c_* = 433 m/min; edge 2).

For experimental cutting speeds, recording the vibration acceleration signals was carried out. Each registered signal was related to a particular tool wear value. The relationship between the vibration acceleration amplitude and the tool wear *A_i_RMS_* = *f*(*VB_C_*) was developed. Graphic presentation of data for cutting speed *v_c_* = 433 m/min is presented in Figure 7 and Figure 8. The root means square value (*RMS*) of vibration acceleration was calculated from the entire frequency band (*f_a_* = 0–20,000 Hz) and placed on the *Y*-axis.

**Figure 9:**
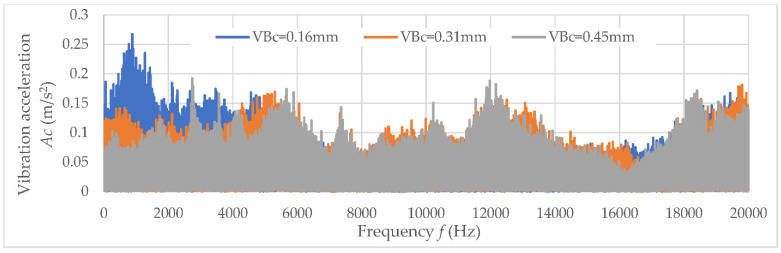
The amplitude-frequency characteristics for main analyzed direction *Ac.*

**Figure 10:**
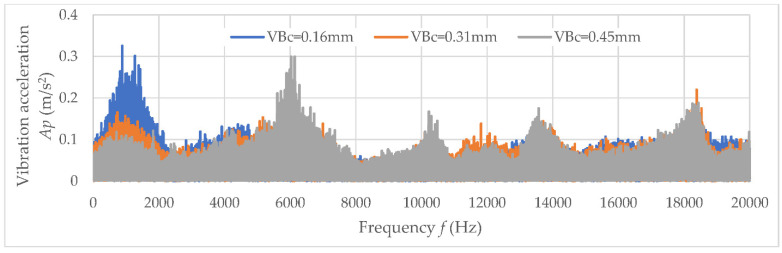
The amplitude-frequency characteristics for main analyzed direction *Ap.*

**Figure 11:**
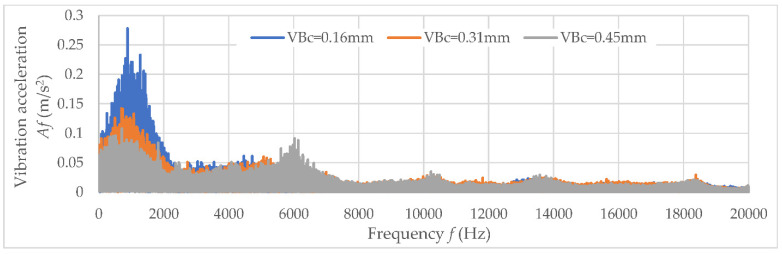
The amplitude-frequency characteristics for main analyzed direction *Af.*

The values of determination coefficient R^2^ and the trend line indicated a lack of correlation between the analyzed data. Therefore, the diagnostic measures were determined in a specific frequency band, which indicates the main correlation to tool wear *VB*_C_. Examples of amplitude-frequency characteristics for three analyzed directions (main *Ac*, feed *Af*, and axial direction *Ap*) are shown in Figure 9, Figure 10 and Figure 11. When the diagnostic measures were designated, the next step was preparing the relevant dataset. Then, based on an appropriate dataset, learning machining to tool wear monitoring following the vibration acceleration signals can be conducted.

The vibration acceleration signals were synchronously sampled in three perpendicular directions in frequency analysis, approx. 25 kHz. The signals were registered for a few seconds, and the total length of the registered time depends on the cutting speed *vc*. Then, the thus obtained data were divided into brief time sections, which were further processed, and teaching data were selected. In making a diagnostic decision model, two tool classes were proposed: usable and unsuitable, and tool corner wear *VBc* = 0.3 mm was assumed as a wear criterion. Table 2 summarizes the information on the available registrations obtained in cutting tests.

**Figure 12:**
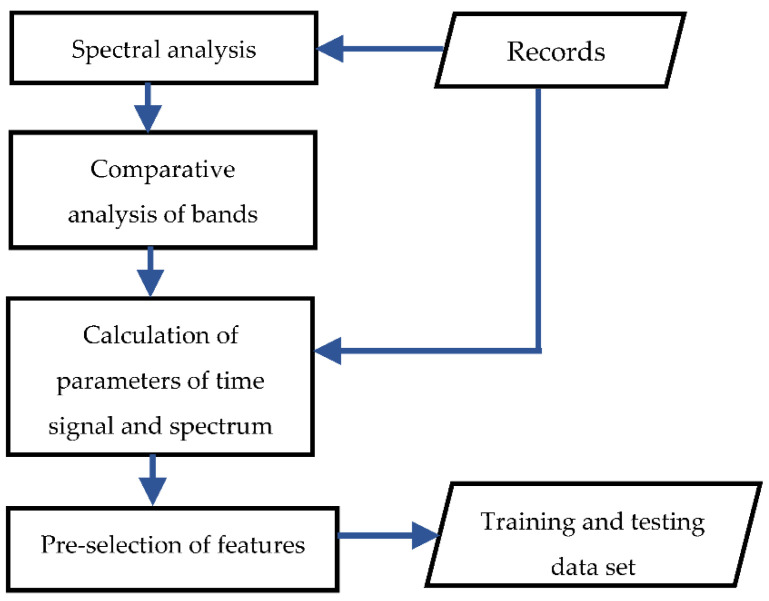
The algorithm for processing the registrations of vibration signals.

In total, 578 registrations and 12,367 teaching examples were obtained. Most of them are examples concerning the usable condition. The tool life is undoubtedly longer than for *v_c_* = 40 m/min at low cutting speeds. Due to the duration of the experiment, the number of examples related to the unsuitable state is relatively lower. Figure 12 depicts the algorithm for processing the obtained registrations.

In the first phase of the research, the analysis of spectrum amplitude connected with minor tool wear (below the tool wear criterion) and extensive tool wear (above the 0.2 mm value) was carried out. Based on comparison analysis, the “active” bands were selected. In these bands, the *RMS* values and the nature of the spectrum showed significant differences for the two tool states. Some “active” bands were only specific to an individual cutting speed and certain to all cutting speeds. The signal measures and parameters characterizing the amplitude spectra were determined in the next step. The values in the entire available band and the filtered signal in the selected bands were determined for the time domain signal.

The following diagnostic measures were determined: root mean square (*RMS*) value, average absolute value, peak signal value, square root amplitude, clearance factor, crest factor, form factor, impulse factor, kurtosis factor, abscissa of signal square, or value of samples exceeding tool criterium concerning root mean square value. Specific new diagnostic measures were identified regarding amplitude spectrum in selected bands. The slenderness ratio (of the spectrum) in the particular band was designated. Based on this ratio, the relevant spectral line in the narrowband can be distinguished from the band without notable frequency. A root mean square value is equally distributed throughout the entire band in such a case. The slenderness ratio was determined as follows:

(2) $$W_{S}=\frac{{w^{\prime }}_{rms}{}^{2}}{w_{rms}{}^{2}}\;.\;$$

where: ${w^{\prime }}_{rms}$. —root mean square value in a narrow window around the maximum of the spectrum in a particular band; $w_{rms}$. —root mean square value in this window. The *RMS* values are calculated directly from the frequency spectrum (square of the amplitude spectrum).

Higher values than 1 indicate the significant spectrum component concerning the overall *RMS* level in the band. For values lower than 1, this component does not exist.

Moreover, the spectrum symmetry ratio in the particular band was designated:

(3) $$W_{Sy}=\frac{{w^{\prime }}_{Lrms}{}^{2}}{w_{Rrms}{}^{2}}.$$

where: ${w^{\prime }}_{Lrms}$. —root mean square value in a narrow window to the left of the maximum spectrum in a particular band; $w_{Rrms}$. —root mean square value in a narrow window to the right of the maximum spectrum in a particular band. The *RMS* values are calculated directly from the frequency spectrum.

As other relevant measures, the frequency coordinate of the spectrum gravity center and the standard deviation of root mean square arrow band were proposed. The main feature of this measure was a regular distribution of individual spectral harmonic components in a narrow band. A total of 350 measures was obtained considering the number of identified bands and three measurement directions.

A classification tree process does not require the pre-selection of features before learning. However, to reduce the number of data and streamline the tree formulation, the evaluation of the diagnostic characteristics was carried out. It also impacted the reduction of features area due to the application of other classification methods.

The principal component analysis (PCA) was not carried out in the current research to obtain easy-to-interpret decision rules. Firstly, the features pre-selection was conducted by comparing the measures of the features separately and visually for the usable and unsuitable conditions. It was established that the lack of noticeable differences in subsets indicated the slight suitability of a particular measure in the classification process. In the next step, the Fisher criterion was applied according to the formula [30]:

(4) $$FE\left(f\right)=\frac{\sum_{j=1}^{C}n_{j}{\left(\mu_{ij}-\mu_{i}\right)}^{2}}{\sum_{j=1}^{C}n_{j}\sigma_{ij}^{2}}\;$$

where: *μ_i_*—average value of feature *f* ; *n_j_*—the number of examples in the class, with index *j; μ_ij_ and σ_ij_*—average value and variance of feature *f* for class *j*; *C*—the number of classes.

This measure is used to evaluate the features that are simultaneously close to the similar value and different in both classes. Moreover, based on the Fisher criterion, the ranking of the features can be determined. An arbitrary limit was adopted in the analysis of the obtained values, and 72 features with relatively high scores were selected. These measures represented feature vector elements in the classification tree. As already indicated, the proper selection of features using a specific quality division measure was specified in the tree-building algorithm. Therefore, it was not necessary to take additional steps to limit the number of features. However, applicating other classification methods, the supplementary selection had to be applied due to having too many features compared to the number of the example. In such a case, the created feature space is not densely filled with examples.

In the next step, a direct measure based on information gain was used, and 15 main measures were selected. Fuzzy rules were induced according to the following algorithm:

- Divide the data set into intervals and subordinate them to membership functions using triangular functions;
- Generate rules for a obtain training example considering the entire memberships of a obtain attribute value in a fuzzy set. Thereby, as many rules are generated as there are learning examples;
- Define the statistical weight of each rule by finding the product of the rule’s predecessors;
- Sort rules by the statistical weight. Remove repeating rules from the set for rules matching the same class. Remove rules with a lower statistical weight for incompatible rules;
- Limit the number of rules by eliminating rules of low statistical weight (the arbitrary limit is 0.7).

**Figure 13:**
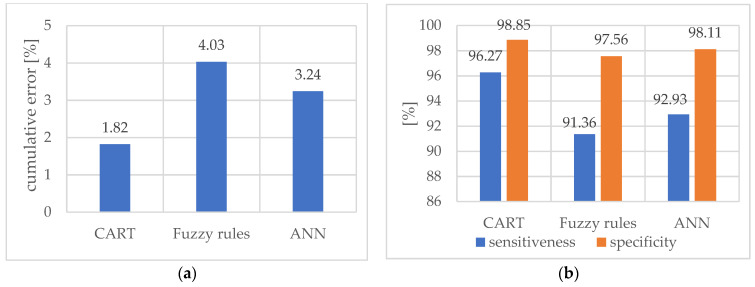
Comparison of cumulative error (**a**), sensitiveness, and specificity (**b**) (considering the cutting parameter *v_c_*).

In the next step, the classification of the test examples using an ordered set of rules was carried out. Table 3 shows the mean results (obtained by 10-fold cross-validation method) of the basic classifier assessments in the form of a tree and direct rule induction. Additionally, the results were compared with the results obtained using a multilayer neural network for pattern recognition with the output softmax layer. Many network structures were tested. The presented results concern the structure with the lowest mean error. In this table, the cutting parameter *vc*, which was one of the attributes of the training examples, was included in this analysis. Finally, Figure 13 depicts a comparison of cumulative error (a), sensitiveness, and specificity (b) for each method in a diagram form.

**Figure 14:**
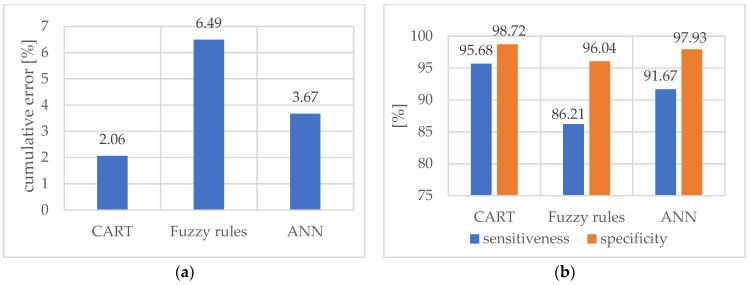
Comparison of cumulative error (**a**), sensitiveness, and specificity (**b**) (without the cutting parameter *v_c_*).

In Table 4, the final results without including the cutting speed *vc* as the output parameter was presented. It was found that considering this parameter as information required for the proper operation of the supervision system may be susceptible to errors in industrial conditions. Therefore, it was decided to check whether the obtained results would be acceptable without information about *vc* available for the classifier. Figure 14 depicts a comparison of cumulative error (a), sensitiveness, and specificity (b) for each method in a diagram form (without the cutting parameter *vc*).

## 3. Discussion

The best results were obtained using neural networks for two hidden layers and 15 and 20 sigmoidal neurons in the layers, including the cutting parameter *vc* as output. However, in the case without considering the cutting speed *vc*, the application of 10 and 12 neurons in hidden layers showed encouraging results.

In the analysis of the results in Table 3 and Table 4, the absence of the cutting parameter *vc* as output increased cumulative errors. In use of the CART method, the error increased only slightly. The analysis of the obtained tree indicated which features were used by the algorithm for its construction. These features are assessed as significant in the division into particular classes. Finally, 39 parameters were selected. The most frequently recurring parameters in the time domain are: form factor, root mean square value, average value, and square root amplitude in different frequency bands (between 5000 and 7000 Hz, from 2500 to 6000 Hz and from 20 to 2000 Hz). Most of the measures apply to the Z measurement direction. However, some necessary measures are simultaneously related to X and Y directions. The omission of these measures resulted in an increase in error by approx. 0.4%. Therefore, all three measurement directions were considered in building the diagnostic system. The primary measure determined in the frequency domain was the root mean square value in a narrow window around the maximum frequency in different frequency bands. A considerable error was obtained using the induced fuzzy rules; thus, this method seems adverse.

## 4. Conclusions

Based on the experimental results, the authors proposed easy intelligent systems for identifying tool wear during turning gray cast-iron EN-GJL-250 using carbide cutting inserts. Due to the ineffective classical regression model and the low correlation coefficient based on vibration acceleration signals, the classification and regression tree, induced fuzzy rules, and artificial neural network were applied. The analyses of the presented research allow us to draw the following conclusions:

- The analysis of the relationship between the vibration acceleration amplitude and the tool wear identified a lack of correlation between the analyzed data. Low coefficient R^2^ values indicate using more complex models than regression.
- The CART model proved to be the most reliable and practical diagnostic supervision system to classify usable/unsuitable tools. Based on this model, the cumulative error was the lowest, especially in analysis without the cutting parameter *v_c_* (2.06%), which seems acceptable for industrial needs.
- The ANN model also had satisfactory results, particularly considering the cutting parameter *v_c_* (3.24%). However, considering this parameter as information required for the proper operation of the diagnostic system may be susceptible to errors in industrial conditions.
- Based on the CART method, the most frequently recurring parameters were also selected: from factor, root mean square value, average value and square root amplitude in different frequency bands in the time domain, and root mean square value in a narrow window around the maximum frequency in different frequency bands in the frequency domain. These signal features have a significant impact on identifying the cutting-edge condition.
- To sum up, using the intelligent system to identify the tool wear during gray cast-iron turning is a relevant prediction tool. In addition, developed models based on input parameters such as cutting speed and vibration acceleration are significant to identifying tool wear’s condition during turning.

## Figures and Tables

**Table 1:** Research plan of grey cast-iron turning.

Cutting Speed *v_c_* (m/min)	Spindle Speed *n* (rev/min)	Feed *f* (mm)	Cutting Depth *a_p_* (mm)
216	265		
314	425	0.1	0.2
433	530		

**Table 2:** Summary of registration and participation of each state class.

Cutting Speed *v_c_*	Number of Tested Tools	Total Number of Complete Registrations	Number of Teaching Examples	Registration Related to the Usable Condition	Registration Related to the Unsuitable Condition
216	2	230	4921	76.5%	23.5%
314	3	243	5200	74%	26%
433	3	105	2246	58%	42%

**Table 3:** Final results of three classification methods (considering the cutting parameter *v_c_*).

Method	Cumulative Error %	Sensitiveness %	Specificity %
Classification Tree CART	1.82	96.27	98.85
Induced Fuzzy Rules	4.03	91.36	97.56
Artificial Neural Network	3.24	92.93	98.11

**Table 4:** Final results of three classification methods (without the cutting parameter *v_c_*).

Method	Cumulative Error %	Sensitiveness %	Specificity %
Classification Tree CART	2.06	95.68	98.72
Induced Fuzzy Rules	6.49	86.21	96.04
Artificial Neural Network	3.67	91.67	97.93

## Data Availability

The data presented in this study are available from the corresponding author, M.W.P., upon reasonable request.

## References

[1] Züfle M., Moog F., Lesch V., Krupitzer C., Kounev S. A machine learning-based workflow for automatic detection of anomalies in machine tools. ISA Trans..

[2] Brillinger M., Wuwer M., Hadi M.A., Haas F. (2021). Energy prediction for CNC machining with machine learning. CIRP J. Manuf. Sci. Technol..

[3] Sika R., Rogalewicz M., Popielarski P., Czarnecka-Komorowska D., Przestacki D., Gawdzińska K., Szymański P. (2020). Decision Support System in the field of defects assessment in the Metal Matrix Composites castings. Materials.

[4] Zhang Y., Xu X. (2021). Machine learning cutting force, surface roughness, and tool life in high speed turning processes. Manuf. Lett..

[5] Aydın K., Akgün A., Yavaş Ç., Gök A., Şeker U. (2021). Experimental and numerical study of cutting force performance of wave form end mills on Gray Cast Iron. Arab. J. Sci. Eng..

[6] Aslan A. (2020). Optimization and analysis of process parameters for flank wear, cutting forces and vibration in turning of AISI 5140: A comprehensive study. Measurement.

[7] Upadhyay V., Jain P.K., Mehta N.K. (2013). In-process prediction of surface roughness in turning of Ti–6Al–4V alloy using cutting parameters and vibration signals. Measurement.

[8] Twardowski P., Tabaszewski M., Wiciak-Pikuła M., Felusiak-Czyryca A. (2021). Identification of tool wear using acoustic emission signal and machine learning methods. Precis. Eng..

[9] Severino G., Paiva E.J., Ferreira J.R., Balestrassi P.P., Paiva A.P. (2012). Development of a Special Geometry Carbide Tool for the Optimization of Vertical Turning of Martensitic Gray Cast Iron Piston Rings. Int. J. Adv. Manuf. Technol..

[10] Tooptong S., Nguyen D., Park K.-H., Kwon P. (2021). Crater wear on multi-layered coated carbide inserts when turning three distinct cast irons. Wear.

[11] Tewary U., Paul D., Mehtani H.K., Bhagavath S., Alankar A., Mohapatra G., Sahay S.S., Panwar A.S., Karagadde S., Samajdar I. (2022). The origin of graphite morphology in cast iron. Acta Mater..

[12] Wang W., Jing T., Gao Y., Qiao G., Zhao X. (2007). Properties of a gray cast iron with oriented graphite flakes. J. Mater. Processing Technol..

[13] Pan S., Zeng F., Su N., Xian Z. (2020). The effect of niobium addition on the microstructure and properties of cast iron used in cylinder head. J. Mater. Res. Technol..

[14] Schultheiss F., Bushlya V., Lenrick F., Johansson D., Kristiansson S., Ståhl J.-E. (2018). Tool Wear Mechanisms of PCBN tooling during High-Speed Machining of Gray Cast Iron. Procedia CIRP.

[15] Tooptong S., Park K.-H., Kwon P. (2018). A comparative investigation on flank wear when turning three cast irons. Tribol. Int..

[16] Luan X., Zhang S., Li J., Mendis G., Zhao F., Sutherland J.W. (2018). Trade-off analysis of tool wear, machining quality and energy efficiency of alloy cast iron milling process. Procedia Manuf..

[17] Chen J., Liu W., Deng X., Wu S. (2016). Tool life and wear mechanism of WC–5TiC–0.5VC–8Co cemented carbides inserts when machining HT250 gray cast iron. Ceram. Int..

[18] Tu G., Wu S., Liu J., Long Y., Wang B. (2016). Cutting performance and wear mechanisms of Sialon ceramic cutting tools at high speed dry turning of gray cast iron. Int. J. Refract. Met. Hard Mater..

[19] Fiorini P., Byrne G. (2016). The influence of built-up layer formation on cutting performance of GG25 grey cast iron. CIRP Ann..

[20] Mohanraj T., Shankar S., Rajasekar R., Sakthivel N.R., Pramanik A. (2020). Tool condition monitoring techniques in milling process—A review. J. Mater. Res. Technol..

[21] Mohanraj T., Yerchuru J., Krishnan H., Nithin Aravind R.S., Yameni R. (2021). Development of tool condition monitoring system in end milling process using wavelet features and Hoelder’s exponent with machine learning algorithms. Measurement.

[22] Zhou Y., Sun B., Sun W. (2020). A tool condition monitoring method based on two-layer angle kernel extreme learning machine and binary differential evolution for milling. Measurement.

[23] Xu G., Zhou H., Chen J. (2018). CNC internal data based incremental cost-sensitive support vector machine method for tool breakage monitoring in end milling. Eng. Appl. Artif. Intell..

[24] Li W., Liu T. (2019). Time varying and condition adaptive hidden Markov model for tool wear state estimation and remaining useful life prediction in micro-milling. Mech. Syst. Signal Process..

[25] Dhobale N., Mulik S., Jegdeeshwaran R., Ganer K. (2021). Multipoint milling tool supervision using artificial neural network approach. Mater. Today Proc..

[26] Laouissi A., Yallese M.A., Belbah A., Belhadi S., Haddad A. (2019). Investigation, modeling, and optimization of cutting parameters in turning of gray cast iron using coated and uncoated silicon nitride ceramic tools. Based on ANN, RSM, and GA optimization. Int. J. Adv. Manuf. Technol..

[27] Herwan J., Kano S., Ryabov O., Sawada H., Kasashima N., Misaka T. (2020). Predicting Surface Roughness of Dry Cut Grey Cast Iron Based on Cutting Parameters and Vibration Signals from Different Sensor Positions in CNC Turning. Int. J. Autom. Technol..

[28] Shirai M., Yamada H. (2020). Mechanical Properties Prediction of Gray Cast Iron Considering Trace Elements Based on Deep Learning. Mater. Trans..

[29] Mills B., Redford A.H. (1983). Machinability of Engineering Materials.

[30] Quanquan G., Li Z., Han J. Generalized fisher score for feature selection. Proceedings of the 27th Conference on Uncertainty in Artificial Intelligence.

